# Genome-Wide Identification and Characterization of the *HAK* Gene Family in Quinoa (*Chenopodium quinoa* Willd.) and Their Expression Profiles under Saline and Alkaline Conditions

**DOI:** 10.3390/plants12213747

**Published:** 2023-11-01

**Authors:** Yanqiong Chen, Yingfeng Lin, Shubiao Zhang, Zhongyuan Lin, Songbiao Chen, Zonghua Wang

**Affiliations:** 1 Fuzhou Institute of Oceanography, Minjiang University, Fuzhou 350108, China; chenyq@mju.edu.cn; 2 Fujian University Engineering Research Center of Marine Biology and Drugs, College of Geography and Oceanography, Minjiang University, Fuzhou 350108, China; 3 College of Agriculture, Fujian Agriculture and Forestry University, Fuzhou 350002, Chinazhangsbiao@aliyun.com (S.Z.)

**Keywords:** *HAK* gene family, quinoa, salt stress, alkali stress, expression profile

## Abstract

The high-affinity K^+^ transporter (HAK) family, the most prominent potassium transporter family in plants, which involves K^+^ transport, plays crucial roles in plant responses to abiotic stresses. However, the *HAK* gene family remains to be characterized in quinoa (*Chenopodium quinoa* Willd.). We explored HAKs in quinoa, identifying 30 members (*CqHAK1*–*CqHAK30*) in four clusters phylogenetically. Uneven distribution was observed across 18 chromosomes. Furthermore, we investigated the proteins’ evolutionary relationships, physicochemical properties, conserved domains and motifs, gene structure, and *cis*-regulatory elements of the CqHAKs family members. Transcription data analysis showed that *CqHAKs* have diverse expression patterns among different tissues and in response to abiotic stresses, including drought, heat, low phosphorus, and salt. The expressional changes of *CqHAKs* in roots were more sensitive in response to abiotic stress than that in shoot apices. Quantitative RT-PCR analysis revealed that under high saline condition, *CqHAK1*, *CqHAK13*, *CqHAK19*, and *CqHAK20* were dramatically induced in leaves; under alkaline condition, *CqHAK1*, *CqHAK13*, *CqHAK19*, and *CqHAK20* were dramatically induced in leaves, and *CqHAK6*, *CqHAK9*, *CqHAK13*, *CqHAK23*, and *CqHAK29* were significantly induced in roots. Our results establish a foundation for further investigation of the functions of *HAKs* in quinoa. It is the first study to identify the HAK gene family in quinoa, which provides potential targets for further functional study and contributes to improving the salt and alkali tolerance in quinoa.

## 1. Introduction

Potassium (K^+^) is the predominant monovalent cation in plant cells, comprising approximately 2–10% of the dry weight of plants [1]. K^+^ is an essential element for plants to maintain normal physiological and biochemical processes. The application of K^+^ fertilizer has been shown to have positive effects on leaf growth [2], flowering [3], wood quality [4], and yield [5]. Plant K^+^ transporters are responsible for K^+^ uptake and transport, and play significant roles in the responses to biotic and abiotic stresses [6,7,8]. Based on their structure and function, K^+^ transporters can be classified into three groups: (1) KT (K^+^ transporter)/HAK (high-affinity K^+^)/KUP (K^+^ uptake), (2) HKT (high-affinity K^+^ transporter), and (3) CPAs (cation–proton antiporters) [9]. Among them, KT/HAK/KUP (HAK hereafter) is the most prominent K^+^ transporter family, with wide distribution in bacteria, fungi, and plants [10].

HAK transporters have been found to play diverse roles in K^+^ uptake and translocation, and function in stress tolerance and osmotic potential regulation [11]. For example, in Arabidopsis, *AtHAK5* is a prominent high-affinity K^+^ uptake transporter that is strongly induced under K^+^ deficiency conditions (external concentration below 10 μM) [12]. *AtKUP7* is crucial for K^+^ uptake in Arabidopsis root and plays a role in transporting K^+^ into the xylem sap, particularly under K^+^-deficient conditions [13]. In rice, *OsHAK5* is involved in transporting K^+^ from roots to shoots. The overexpression of *OsHAK5* led to increased shoot K^+^/Na^+^ ratio and salt tolerance in rice [14]. Similarly, rice plants overexpressing *OsHAK1* displayed enhanced salt tolerance and significantly improved drought resistance, which resulted an increase in grain yield by 35% compared to the wild type under drought conditions [15,16]. In maize, *ZmHAK4* functions in mediating shoot Na^+^ exclusion, thereby playing a role in promoting salt tolerance [17]. Some HAK transporters have been found to function in the morphological development of roots and shoots. For example, the mutation of *AtKUP2*/*SHY3* (short hypocotyl 3) impacted cell expansion and resulted in developmental defects in Arabidopsis shoots [18]. *AtKUP4*, previously known as tiny root hair 1 (*TRH1*), has been characterized to be involved in the initiation and formation of root hairs [19,20].

Quinoa (*Chenopodium quinoa* Willd.) is an allotetraploid annual halophyte crop (2n = 4x = 36), that originates from the hybridization between diploid *C. pallidicaule* and diploid *C. suecicum* [21]. Quinoa is highly tolerant to multiple abiotic stresses, such as cold, drought, salt stress, and sterile soil [22]. Meanwhile, it possesses remarkable nutritional properties and has emerged as an attractive pseudo-cereal over the past decades. Although the HAK family is the most prominent K^+^ transporter family and has crucial functions in development and in the responses to stresses in plants, very little is known regarding the information of HAKs in quinoa. In the present study, we performed a genome-wide identification and characterization of the *HAK* gene family in quinoa. Our analyses revealed the phylogenetic relationships, conserved motifs and domains, gene structure, *cis*-acting elements, syntenic relationships, tissue expression patterns, and the salinity and alkalinity-induced expression profiles of the *HAK* family. By understanding the involvement of the HAK gene family in quinoa’s response to salt and alkali stress, this research seeks to provide valuable insights into the molecular mechanisms underlying quinoa’s resilience and contribute to the development of stress-tolerant varieties.

## 2. Results

### 2.1. Identification of the HAK Family Genes in C. quinoa

A total of 30 *CqHAK* genes were determined (Table 1) by searching the quinoa genome [21]. Table 1 presents the basic properties of the 30 CqHAK members, including number of introns, length, molecular weight (MW), isoelectric point (pI), and the putative subcellular localization of predicted proteins. The *CqHAK* genes encode proteins with lengths ranging from 476 to 910 amino acids. The genomic sequences of CqHAK contained 4 to 13 exons. The pI of the predicted CqHAK proteins ranged from 6.1 to 9.17, and the theoretical MW ranged from 54.19 to 102.23 KDa. Subcellular location analyses indicated that all CqHAKs were predicted to be located in the plasma membrane.

### 2.2. Phylogenetic Analysis of the CqHAK Transporters

**Figure 1:**
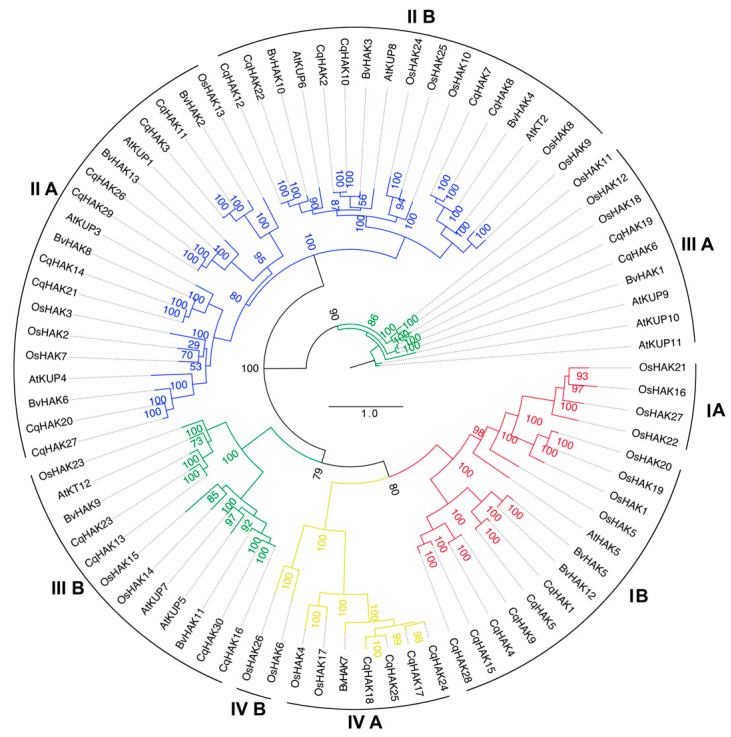
Phylogenetic analysis of the *HAK* families in quinoa, *Arabidopsis thaliana*, *Beta vulgaris* and *Oryza sativa*. The phylogenetic tree was constructed using the IQ tree. Four clusters (I, II, III, IV) were labeled as red, blue, green and yellow respectively.

The 30 identified CqHAKs were classified into four clusters: I, II, III, and IV (Figure 1). Furthermore, each cluster could be subdivided into sub-clades A and B. Cluster I contained six CqHAKs, with all six clustering in IB. Cluster II consisted of 14 CqHAKs, with eight members in IIA (CqHAK3, 11, 14, 20, 21, 26, 27, and 29) and six in IIB (CqHAK2, 7, 8, 10, 12, and 22). There were six CqHAKs in cluster III, with two members in IIIA (CqHAK6 and 19) and four in IIIB (CqHAK13, 16, 23, and 30). Only four CqHAK transporters were categorized into Cluster IV, with all (CqHAK17, 18, 24, and 25) clustering together in IVA.

### 2.3. Motif, Domain, and Gene Structure Analyses of CqHAKs

**Figure 2:**
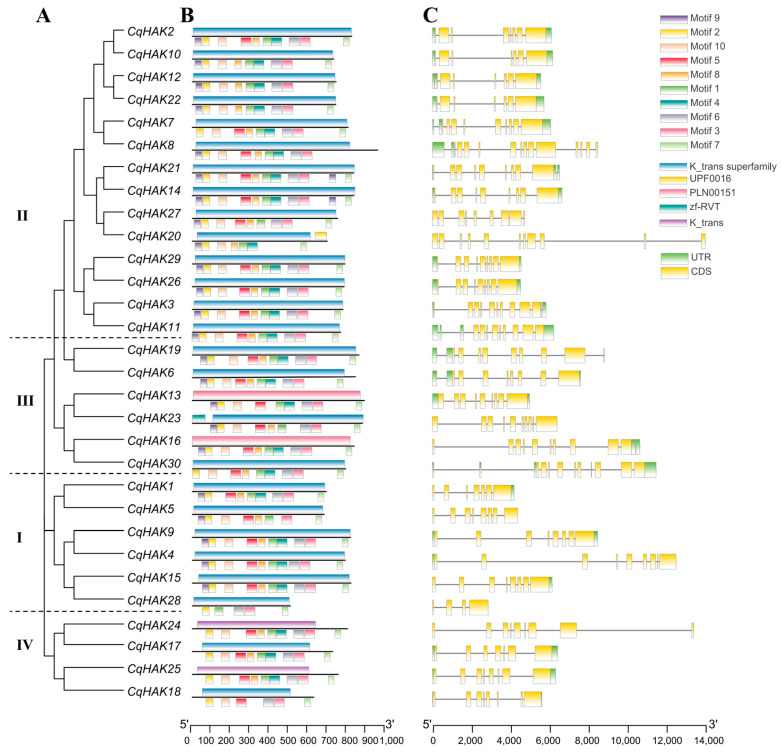
Phylogenetic relationships, gene structure, and motifs of the *HAK* genes in *C. quinoa* using TBtools. (**A**) The ML method constructed the phylogenetic tree based on the full-length sequences of the CqHAK proteins. (**B**) The motif and domain composition of the CqHAK proteins. The conserved domains and motifs were indicated on the protein sequences’ upper and lower sides, respectively. (**C**) Exon–intron structures of the *CqHAK* genes. Blue–green boxes indicate untranslated 5′- and 3′- regions, yellow boxes indicate exons, and black lines indicate introns.

Ten distinct motifs (labeled as Motif 1–10) were identified among the CqHAK family members (Figure 2A,B and Figure S1). Most of the CqHAK members contained more than seven motifs, except for CqHAK18 and CqHAK28, which contained only six and five motifs, respectively. Specifically, Motif 2 was observed in all the CqHAK proteins. Motifs 1, 3, and 7 were conserved in all four HAK clusters. Motif 9 had the highest absence frequency, as it was missing in seven HAKs. Motifs 4 and 5 had the second highest absence frequency, as it was missing in five HAKs. 

The HAK family members have a specific domain known as the “K_trans superfamily” (cl15781). Based on NCBI conserved domain database, there are eight types of “K_trans superfamily”. Among these, three were found in the CqHAK members, namely “K_trans,” “PLN00151,” and “K_trans superfamily”. Cluster I and Cluster II contained only the “K_trans superfamily,” while clusters III and IV contained the “K_trans superfamily” along with “PLN00151” and “K_trans,”, respectively (Figure 2A,B).

The gene structures of *CqHAKs* exhibited significant variability, comprising four to 13 exons and three to 12 introns (Figure 2C). Eleven *CqHAK* genes (36.7%) have nine introns, seven *CqHAK* genes have seven introns, five *CqHAK* genes have six introns, four CqHAK genes have nine introns, and the remaining three *CqHAK* genes contain three, ten and 12 introns each. The number of exons/introns was similar in *CqHAKs* that were categorized into same cluster, especially in the phylogenetically closest pairs. For instance, the gene pairs *CqHAK12* and *CqHAK22*, *CqHAK14* and *CqHAK21*, *CqHAK16* and *CqHAK30*, *CqHAK26* and *CqHAK29* contained the same intron numbers, respectively. The exon configuration of most gene pairs was almost the same regardless of the difference in intron length. However, the gene pairs of *CqHAK17* and *CqHAK24*, and *CqHAK20* and *CqHAK27* showed obvious difference in gene structure (Figure 2C).

### 2.4. Chromosomal Distribution and Synteny Analysis of the CqHAK Genes

**Figure 3:**
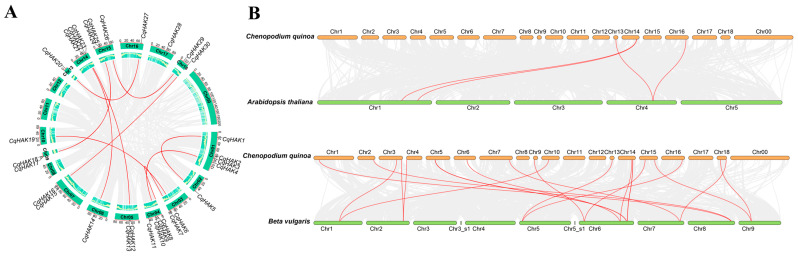
Chromosome distributions of the *HAK* genes in *C. quinoa* using TBtools. (**A**) The chromosomal location and interchromosomal relationship of the *HAK* genes in *C. quinoa*. (**B**) Synteny analysis of the *HAK* genes between *C. quinoa* and *A. thaliana*, and *C. quinoa* and *B. vulgaris*. Gray lines in the background indicate the collinear blocks, and the red lines highlight the syntenic *HAK* gene pairs.

The genomic distribution of the *HAK* genes in quinoa was identified by mapping the ORFs of all determined genes onto their corresponding chromosome (Figure 3A). The distribution of the *HAK* genes in the quinoa chromosomes was uneven, with no genes identified on chr00, chr8, chr11, nor chr12. Chr1 and chr4 had the highest gene count, containing four genes. Chr14 and chr15 each contained three genes, while chr3, chr5, chr7, chr9, and chr18 each contained two genes. Chr2, chr6, chr10, chr13, chr16, and chr17 had only one gene distributed.

A synteny analysis was conducted to further explore the duplication event that occurred in the *CqHAK* gene family. There were 22 *CqHAKs* with a collinear relationship (Figure 3A). Only two pairs of tandem repeat events, *CqHAK17*/*CqHAK18* and *CqHAK24*/*CqHAK25* were found in the quinoa genome. In addition, 13 segmental duplication events involving 20 *CqHAK* genes were identified (Table S2). Notably, four and 18 orthologous pairs were found between quinoa and *A. thaliana*, quinoa and *B. vulgaris*, respectively (Figure 3B). These genes may play an irreplaceable role in the evolution of the HAK family. The nonsynonymous (Ka) and synonymous (Ks) substitution rates were calculated to investigate the evolutionary selection pressure in forming the HAK gene family. The Ka/Ks ratios of 33 *HAK* gene pairs were analyzed to compare Cq-Cq, Cq-At, and Cq-Bv (Figure S1, Table S3). All gene pairs’ Ka/Ks ratios were below 1.00, suggesting that the HAK genes may have experienced significant purification selection pressure throughout the quinoa evolution.

### 2.5. Cis-Regulatory Elements Analysis of the CqHAK Genes

**Figure 4:**
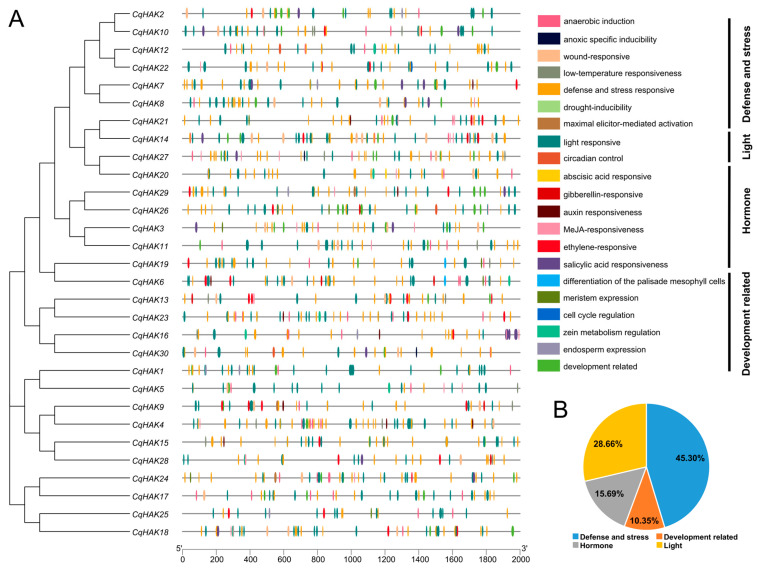
Putative *cis*-acting elements and transcription factor binding sites in the promoter regions of the *HAK* genes in *C. quinoa* (**A**), and four functional types of *cis*-acting elements and their proportion in all the *CqHAKs* genes (**B**).

To elucidate the signal transduction pathway of *CqHAKs*, we searched the 2 kb upstream sequences of the *CqHAK* genes for candidate *cis*-element analysis. A total of 1256 *cis*-acting elements were determined. Based on their functional annotations, these *cis*-elements could be categorized into four groups: (1) those associated with defense and stress (e.g., low-temperature, drought, wounding, and hypoxia); (2) those related to development (e.g., endosperm, meristem, palisade mesophyll cells, and cell cycle regulation); (3) those involved in the responses to plant hormones (e.g., ABA, MeJA, GA, auxin, salicylic acid, and ethylene); and (4) light-responsive element (Figure 4A). The most frequent *cis*-element in the promoters of the *CqHAK* genes were associated with defense and stress (Figure 4B).

### 2.6. Expression Pattern Analysis of the CqHAK Genes in Various Tissues and under Abiotic Stresses

**Figure 5:**
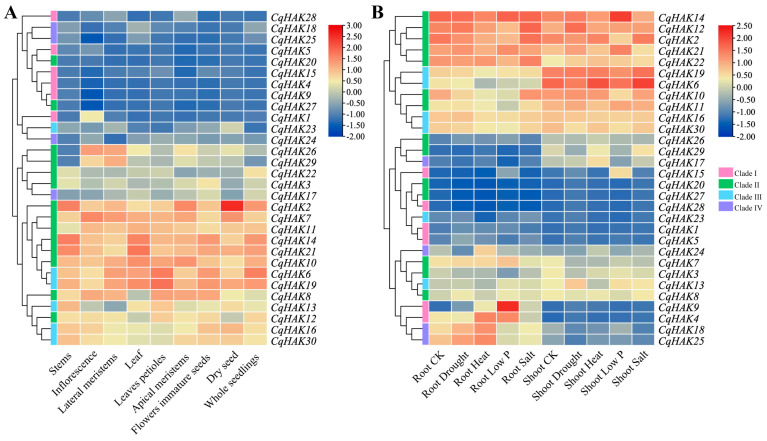
Heatmap of *HAK* gene expression in different tissues (**A**) and treatments in the quinoa roots and shoots (**B**). CK represented as blank control group, where quinoa was grown in soil without any treatment.

We analyzed the expression patterns of the *CqHAK* genes in different tissues using publicly available transcriptome data (Figure 5A, Table S4). Certain *CqHAK* genes showed similar expression patterns, while others displayed significant tissue-specific expression patterns, indicating the functional divergence of *CqHAKs* among different tissues during different developmental stages. Minimal expression levels of *CqHAK4*, *CqHAK9*, *CqHAK15*, *CqHAK20*, *CqHAK25*, and *CqHAK27* were observed across various tissues. The consistently high expression of *CqHAK2*, *CqHAK6*, *CqHAK7*, *CqHAK10*, *CqHAK14*, *CqHAK19* and *CqHAK21* were observed in all tissues. Certain genes exhibit tissue specificity. For instance, *CqHAK1* is predominantly expressed in inflorescence. A number of gene pairs (7/11) with high collinearity exhibited similar expression patterns, including *CqHAK1*/*CqHAK5*, *CqHAK4*/*CqHAK9*, *CqHAK6*/*CqHAK19*, *CqHAK14*/*CqHAK21*, *CqHAK16*/*CqHAK30*, *CqHAK20*/*CqHAK27*, and *CqHAK26*/*CqHAK29* (Figure 5A).

The expression levels of the *CqHAK* genes in both root and shoot apices under drought, heat, low phosphorus, and salt stresses were also evaluated (Figure 5B, Table S5). The expression changes of *CqHAKs* were more sensitive in roots in response to abiotic stress than that in shoot apices. *CqHAK20*, *27,* and *28* were expressed at deficient levels in all treatments. In contrast, *CqHAK2*, *12*, *14*, *21,* and *22* exhibited consistently high expression levels under all abiotic stresses. Notably, *CqHAK9* was induced in response to different stress treatments in quinoa roots.

### 2.7. Validation of the Expression Profiles of the HAK Genes under Salt and Alkali Stress by Quantitative RT-PCR (qRT-PCR)

The transcript levels of 25 selected *CqHAKs* in the roots and leaves of the quinoa seedlings exposed to salt stress and alkali stress were measured using qRT-PCR. The results showed that the CqHAK members were expressed in diverse levels and patterns.

**Figure 6:**
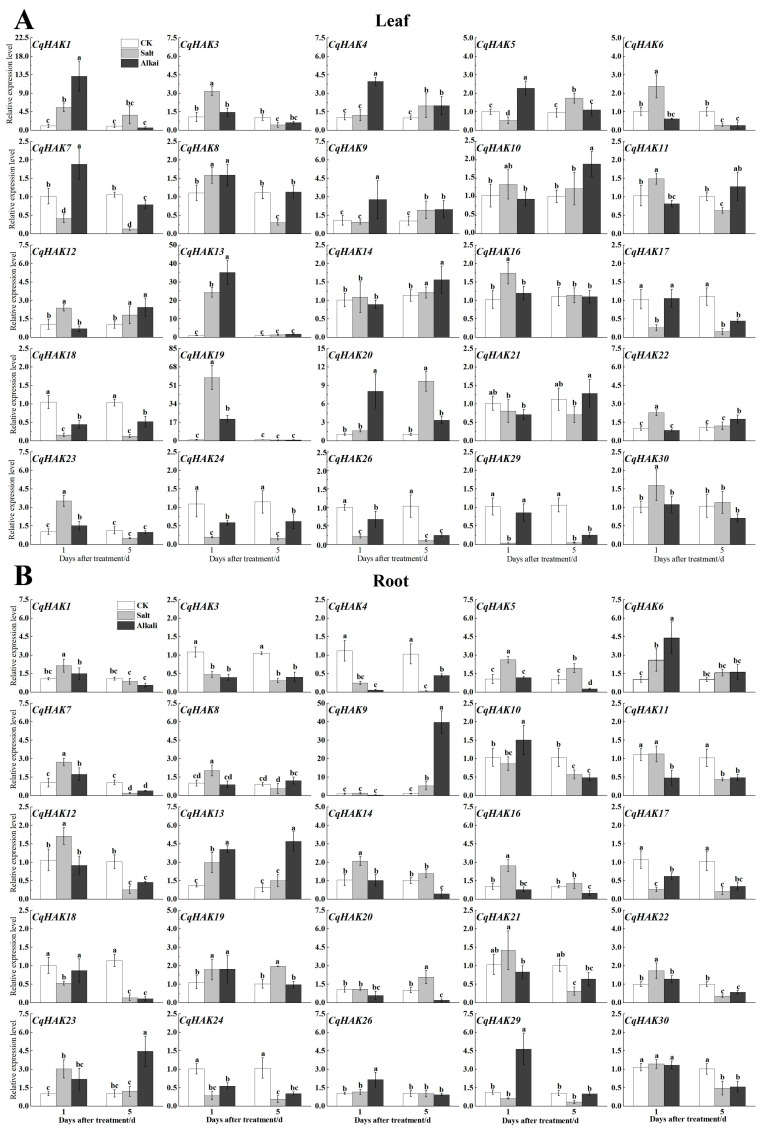
Expression profiling of the *CqHAK* genes under salt (300 mM NaCl) and alkali (40 mM Na_2_CO_3_ and NaHCO_3_ mixture, with mole ratio = 1:2, pH = 9.38) stresses, respectively, in the quinoa roots (**A**) and leaves (**B**) at the seedling stage. The expression levels of *CqTUB*-9 was used to normalize the expression levels of the *CqHAK* genes. CK represent the treatment of quinoa seedlings with 1/2 Hogland nutrient solution. The data are the mean ± SEM of three independent biological samples, and the vertical bar represents the standard error of the mean. Lowercase letters indicated the significant difference at *p*  <  0.05.

Upon salt treatment, the expression of *CqHAK13* and *CqHAK19* was observed to be dramatically induced in leaves at 1 day after treatment (DAT), and the expression of *CqHAK20* was observed to be dramatically induced in leaf at 5 DAT (Figure 6A). Only *CqHAK9* was found to be induced up to 5 fold in root at 5 DAT(Figure 6B). Upon alkali treatment, the expression of *CqHAK1*, *CqHAK13*, *CqHAK19*, and *CqHAK20* was observed to be dramatically induced in leaves at 1 DAT (Figure 6A). Notably, *CqHAK6* (4.3-fold) and *CqHAK29* (4-fold) were significantly induced in the roots at 1 DAT, and *CqHAK9* (37-fold), *CqHAK13* (5-fold), and *CqHAK23* (4.3-fold) were significantly induced in root at 5 DAT (Figure 6B). 

## 3. Discussion

The HAK family of the K^+^ transporters have been extensively studied for its role in K^+^ transport across membrane in bacteria, fungi, and plants [11]. HAK plays a significant role in catalyzing K^+^ acquisition and uptake, and maintaining plant cation homeostasis, thereby contributing to plant growth and development [23,24,25]. In this study, 30 HAK family members were identified in the quinoa genome. This family has been found in different plant species: 6 *HAK* genes in *Amborella trichopoda*,13 *AtHAK* in Arabidopsis, 27 *OsHAK* in rice, 40 *BnHAK* in *Brassica napus* [26] and 56 *TaHAK* in wheat *Triticum aestivum* [27]. The numbers of the *HAK* genes vary among different plant species, which could be due to ancient whole-genome duplication (WGD), segmental duplication (SD) and tandem duplication (TD) [28,29]. Quinoa experienced a WGD of approximately 4.3 Mya [30], which may contribute to the rapid gene expansion of the *CqHAK* family. Additionally, we identified four *CqHAK* genes clustered into two tandem duplication events and ten segmental duplication events (Table S2). Intraspecies duplication events (SD, 13.33%; TD, 66.67%) were the main contributors to quinoa’s rapid gene expansion of the *HAK* family. These findings suggest that *CqHAKs* underwent species-specific expansion during long-term evolution.

After identifying the conserved domains, all CqHAK members were confirmed to contain HAK domains. Consistent with the findings in other plant species, the CqHAK proteins are highly conserved in the length of amino acid. Previous study has indicated that the *AtHAK* and *OsHAK* members could be classified into four clusters (clusters I to IV) [31]. Based on these criteria, *CqHAKs* were also classified into the same four groups (Figure 1), indicating that the taxonomy and evolution of the *HAK* gene family were highly conserved among different plant species. 

Gene structure (intron–exon structure) is a typical evolutionary marker in gene families [32,33]. The *HAK* gene family exhibited a low degree of conservation in terms of intron–exon structure. The coding sequences of all *CqHAK* genes are disrupted by introns, with the number of introns ranging from three to twelve, suggesting that the gene structure in quinoa is more diverse than that in other model plants, such as Arabidopsis, which typically have five to nine introns [34], and rice, which typically have one to nine introns within the *HAK* genes [31]. Only four (4/11) homologous *CqHAK* gene pairs shared a similar gene structure; *CqHAK12*/*CqHAK22* had six introns, while *CqHAK14*/CqHAK21 and *CqHAK26*/*CqHAK29* contained eight introns each, and *CqHAK16*/*CqHAK30* had nine introns. (Figure 2, Table 1). This phenomenon indicates that intron acquisition or loss may have occurred in the *HAK* gene family during evolution, leading to the emergence of homologous genes with diverse structures.

*Cis*-acting regulatory elements play a pivotal role in transcriptional regulation in various biological processes [35] and are significant for plant defense against different biotic and abiotic stresses [36]. In this study, the most frequent predicted *cis*-elements in the promoters of the *CqHAK* genes were associated with defense and stress responses, such as BOX4 (light-responsive), ABRE (abscisic acid-responsive), ARE (anaerobic induction), TGACG and TGACG (MeJA-responsive), and STRE (stress-responsive) (Figure 4B). These *cis*-elements may play important roles in regulating expression of genes in response to these biotic and abiotic stresses.

The expression pattern can be closely associated with gene function. Our study revealed that the expression levels of most of the *CqHAK* genes in Clade I and Clade IV were relatively low across in all the organs and under diverse abiotic stress treatments. In contrast, most of the *CqHAK* genes in Clade II and Clade III showed higher expression levels in all test tissues (Figure 5). Similar results were observed in rice [31], Arabidopsis [34], wheat [27], and Saccharum [29].

Soil saline-alkalization has emerged as a significant and escalating global issue [37]. The extensive abiotic stressor severely hampers crop production and threatens agriculture and food security worldwide [38,39]. Quinoa is an excellent pseudocereal for utilization in saline–alkaline environments due to its inherent resistance to salt and alkali. However, the mechanism underlying its salt and alkali tolerance still needs to be better understood. Compared to saline stress, alkaline stress has been found to cause more severe disruptions in trophic ion regulation, osmotic balance, antioxidant defense systems, and plant growth [40]. Alkaline stress has a more substantial effect on K^+^/Na^+^ homeostasis than saline stress in many plants. The K^+^ concentration is related to regulating osmosis, membrane potential, and enzyme activity in plants [7]. Previous studies have demonstrated that the *HAK* family members *AtHAK5*, *AtKUP7* and *OsHAK1* mediated K^+^ acquisition, thereby enhancing high-affinity K^+^ uptake in Arabidopsis and rice [11,12,13,41,42,43]. 

This study showed that some *CqHAKs* were affected by saline and alkali stresses. For instance, *CqHAK9* was induced in leaf of quinoa upon alkali treatments; *CqHAK1*, *CqHAK13*, *CqHAK19*, and *CqHAK20* were induced in leaf of quinoa upon both salt and alkali treatments; *CqHAK6*, *CqHAK9*, *CqHAK13*, *CqHAK23*, and *CqHAK29* were induced in the roots of quinoa upon alkali treatment; and *CqHAK13* was induced upon both salt and alkali treatment in both leaf and root of quinoa. These results suggested that these genes may play important but distinct roles in quinoa in response to saline and alkali stresses. Further research would be required to elucidate the precise role of the *CqHAK* genes in quinoa.

## 4. Summary

In summary, 30 *HAK* genes were identified in *C. quinoa*. Phylogenetic analysis indicated that these HAK proteins were classified into four groups. The *CqHAK* gene family was investigated by evaluating the gene structure, chromosomal distribution, synteny, *cis*-acting regulatory element, and expression patterns. The expression of the *CqHAK* genes varies in different tissues and upon abiotic treatments. qRT-PCR further verified that the expression profiles of *CqHAKs*. Specifically, *CqHAK13* was the only gene which highly induced under saline and alkaline treatment both in leaf and root of quinoa. Upon saline condition, four genes (*CqHAK1*, *CqHAK13*, *CqHAK19*, and *CqHAK20*) were dramatically induced in leaf; under alkaline condition, five genes in leaf (*CqHAK1*, *CqHAK9*, *CqHAK13*, *CqHAK19*, and *CqHAK20*) and five genes in root (*CqHAK6*, *CqHAK9*, *CqHAK13*, *CqHAK23*, and *CqHAK29*) were significantly induced. Our results offer essential insights into the HAK family in quinoa, providing a solid foundation for future investigation of the functions of *HAKs* in quinoa.

## 5. Materials and Methods

### 5.1. Identification of HAK Family Members in Quinoa

The genomic data of *C. quinoa* (Cq P1614886 genome V1 pseudomolecule) were obtained from the website (https://www.cbrc.kaust.edu.sa/chenopodiumdb/ (accessed on 29 August 2022)) [21]. Firstly, we conducted two BLASTp searches with a score value ≥ 100 and an E-value ≤ 1–10 using the deduced amino acid sequences of Arabidopsis [44] and rice [31] to pre-screen the candidate HAK protein sequences. Arabidopsis and rice *HAK* gene sequences were obtained from the Plant TAIR database (https://www.arabidopsis.org/ (accessed on 29 August 2022)) and the Ensembl Plants database (http://plants.ensembl.org/ (accessed on 29 August 2022). Subsequently, the pre-screened biomolecular structure was submitted to the NCBI-Swiss-Prot database (http://plants.ensembl.org/ (accessed on 29 August 2022)) for homology comparison, eliminating the closed-source sequences of quinoa HAKs and retaining the non-redundant protein sequences. Finally, the putative HAK sequences were submitted to InterProScan (https://www.ebi.ac.uk/interpro/search/sequence-search (accessed on 29 August 2022)), CDD v3.19 (https://www.ncbi.nlm.nih.gov/Structure/bwrpsb/bwrpsb.cgi (accessed on 29 August 2022)), Pfam (https://pfam.xfam.org/ (accessed on 29 August 2022)), and SMART v9.0 (http://smart.embl-heidelberg.de/ (accessed on 29 August 2022)) to verify the accuracy of the candidate genes. All predicted protein sequences were manually curated using the FGENESH at Softberry website (http://linux1.softberry.com/berry.phtm (accessed on 29 August 2022)). A total of 30 *CqHAK* genes were identified and assigned locations on the chromosome.

### 5.2. Analyses of Sequence, Conserved Motif and Structural Characterization

The fundamental physical and chemical properties of the CqHAK protein sequences were predicted utilizing the ExPASy tool (https://web.expasy.org/compute_pi/ (accessed on 23 December 2022)), including amino acid numbers, isoelectric point and hydrophobicity. The subcellular location of the CqHAK proteins were predicted using the Softberry website. The conserved motifs in the CqHAK proteins were determined and compared to assess the difference using the MEME program v5.5.0 (http://meme-suite.org/tools/meme (accessed on 23 December 2022)) [45]. Sequence structures, including exon–intron positions and conserved motifs, were visualized using TBtools software v2.019 [46].

### 5.3. Analyses of Phylogenetic Relationship

The HAK protein sequences of *A. thaliana*, *O. sativa*, and *B. vulgaris* were acquired from Phytozome (https://phytozome-next.jgi.doe.gov/ (accessed on 29 August 2022)). Multiple sequence alignment and phylogenetic analyses were performed using TBtools [46]. The unrooted phylogenetic tree was generated using the IQ-tree v1.6.12 [47], with 1000 bootstrap replicates and default parameters.

### 5.4. Analysis of Chromosome Locations and Collinearity

The position information of the *HAK* genes was extracted from the GFF annotation file. TBtools was implemented to visualize the chromosome locations of each gene. The collinearity blocks of the *CqHAKs* genes in the entire genome were identified using MCSCAN (Python version) [48]. Interspecific synteny relationships (*C. quinoa* and *A. thaliana*, *C. quinoa*, and *B. vulgaris*) were analyzed and mapped using the Dual Synteny Plotter in TBtools. The substitution rates of synonymous (Ks) and nonsynonymous (Ka) were evaluated utilizing TBtools.

### 5.5. Analysis of Expression Level of HAK Genes

The expression data for various tissues and organs of quinoa (No: PRJNA394651) and different treatments (drought, heat, salt, and low P) (No: PRJNA306026) were obtained from the NCBI BioProject database (www.ncbi.nlm.nih.gov/bioproject (accessed on 28 January 2023)). The control condition (CK) involved cultivation in well-watered soil at a temperature of 20 °C with a 12 h daily light for three weeks. For the heat treatment, the plants were then grown in 12 h light at 37 °C and 12 h dark at 32 °C for one week. The salt treatment involved the use of a 300 mM NaCl solution under hydroponic conditions. The drought treatment entailed withholding water for one week under a temperature of 20 °C and a 12 h daily light. The low phosphorus condition involved cultivation in a medium lacking KH_2_PO_4_. All treatments were carried out for one week. At the end of the cultivation period, root and shoot tissues were harvested and stored in liquid nitrogen for subsequent RNA extraction, and three independent replicates were conducted for each treatment. The data were mapped individually to the *C. quinoa* genome using HISAT2 v2.2.1 (https://github.com/DaehwanKimLab/hisat2 (accessed on 29 August 2023)) [49] with the default parameters. Transcript abundance and differential gene expression were estimated using Cufflink v2.2.1 (https://github.com/cole-trapnell-lab/cufflinks (accessed on 29 August 2023)). The expression levels of the *CqHAK* genes were determined as fragments per kilobase of exon model per million mapped reads (FPKM). A heatmap was generated using TBtools with log_2_(FPKM + 1).

### 5.6. Plant Materials and Treatments

Quinoa seeds (“Jiaqi 744,” obtained from Shanxi Nonggu Jiaqi Seed Industry Co., Ltd., Shanxi, China, http://jqseeds.com/ (accessed on 7 June 2022)) were germinated in the soil until they reached the four-leaf stage, after which they were transferred to a 1/2 Hoagland nutrient solution. Quinoa seedlings were planted in a growth chamber with a photoperiod of 14 h per day and a temperature of 25 ± 1 °C. Upon the full expansion of the eighth leaf, seedlings were transferred to three different culture media: 1/2 Hogland nutrient solution (CK), and 1/2 Hogland nutrient solution supplemented with 300 mM NaCl, and 40 mM of an alkali solution (Na_2_CO_3_ and NaHCO_3_ mixture, with mole ratio = 1:2, pH = 9.38), respectively. The roots and leaves were collected for each treatment at 1 and 5 days. The samples were immediately frozen in liquid nitrogen and stored at −80 ℃ for subsequent analysis.

### 5.7. RNA Extraction, Analysis of the Gene Expression by RT-qPCR, and Correlation Analyses

Total RNA was extracted utilizing the Trizol method (Trizol, TransGen Biotech, Beijing, China), and the first cDNA strand was synthesized utilizing a cDNA first-strand synthesis kit (HiScript^®^ II 1st Strand cDNA Synthesis Kit, R211, Vazyme Biotech, Nanjing, China). The RT-qPCR reaction was conducted using CFX Connecte Thermal Cycler Real-Time PCR system (Bio-Rad, Hercules, CA, USA) with the ChamQ Universal SYBR qPCR Master Mix (Q711, Vazyme Biotech, Nanjing, China). *CqTUB*-9 was utilized as an internal reference gene. The primer sequences utilized in this study are presented in Table S1. The reaction system is 10 μL: 1 μL cDNA, 0.5 μL of the upstream and downstream primers, 5 μL of SYBR, and 3 μL of ddH_2_O. The reaction program is as follows: 95 °C pre-denaturation for 30 s, 95 °C denaturation for 5 s, and 60 °C annealing for 30 s, 40 cycles, repeat 3 times. After the reaction, the fluorescence value change curve and melting curve are analyzed. The relative expression levels of genes were computed utilizing the 2^−ΔΔCt^ method.

## Figures and Tables

**Table 1:** Properties of the predicted HAK proteins in *C. quinoa*.

Name	Gene ID	Length (aa)	Intron	Molecular Weight (Da)	Theoretical PI	Subcellular Localization	Location
*CqHAK1*	AUR62021456	650	6	72,965.38	8.18	Plasma membrane	Chr01: 18278493–18282659
*CqHAK2*	AUR62042411	774	7	87,036.04	6.74	Plasma membrane	Chr01: 88160907–88166983
*CqHAK3*	AUR62040746	731	9	81,227.69	8.83	Plasma membrane	Chr01: 90527306–90533106
*CqHAK4*	AUR62028032	742	8	83,030.02	7.32	Plasma membrane	Chr01: 104924582–104937024
*CqHAK5*	AUR62010943	640	7	71,619.97	8.21	Plasma membrane	Chr02: 54650085–54654440
*CqHAK6*	AUR62034910	744	7	83,398.35	6.78	Plasma membrane	Chr03: 61032690–61040255
*CqHAK7*	AUR62012363	756	8	84,438.67	6.74	Plasma membrane	Chr03: 77154455–77160490
*CqHAK8*	AUR62031491	910	12	10,2229.37	6.93	Plasma membrane	Chr04: 71229–79662
*CqHAK9*	AUR62020693	768	7	86,037.89	7.88	Plasma membrane	Chr04: 17190576–17199015
*CqHAK10*	AUR62035954	687	6	77,129.18	6.48	Plasma membrane	Chr04: 21795245–21801387
*CqHAK11*	AUR62026895	720	8	80,136.43	8.96	Plasma membrane	Chr04: 45774326–45780518
*CqHAK12*	AUR62014006	698	6	77,969.08	6.91	Plasma membrane	Chr05: 31254125–31259655
*CqHAK13*	AUR62014007	835	8	92,889.21	6.22	Plasma membrane	Chr05: 31289460–31294418
*CqHAK14*	AUR62017474	784	8	87,191.16	8.7	Plasma membrane	Chr06: 44873117–44879731
*CqHAK15*	AUR62033186	770	7	86,317.46	6.4	Plasma membrane	Chr07: 95200544–95206655
*CqHAK16*	AUR62001622	786	9	88,068.4	7.88	Plasma membrane	Chr07: 102724390–102734985
*CqHAK17*	AUR62003489	681	6	75,594.81	8.66	Plasma membrane	Chr09: 2936029–2942420
*CqHAK18*	AUR62003490	590	7	65,465.15	6.1	Plasma membrane	Chr09: 2945466–2951051
*CqHAK19*	AUR62026619	809	8	90,741.81	7.88	Plasma membrane	Chr10: 23716177–23724950
*CqHAK20*	AUR62010772	655	10	72,550.63	9.17	Plasma membrane	Chr13: 7774288–7788227
*CqHAK21*	AUR62042858	784	8	87,071.95	8.56	Plasma membrane	Chr14: 46675721–46682205
*CqHAK22*	AUR62005353	696	6	77,683.74	7.08	Plasma membrane	Chr14: 50083331–50089025
*CqHAK23*	AUR62005354	826	9	91,509.16	6.43	Plasma membrane	Chr14: 50115909–50122274
*CqHAK24*	AUR62017798	754	8	83,549.36	8.45	Plasma membrane	Chr15: 19349048–19362387
*CqHAK25*	AUR62017799	708	7	78,514.06	6.52	Plasma membrane	Chr15: 19364004–19370291
*CqHAK26*	AUR62025122	737	8	81,976.91	6.92	Plasma membrane	Chr15: 52273038–52277536
*CqHAK27*	AUR62019774	706	8	79,121.86	9.15	Plasma membrane	Chr16: 70971581–70976287
*CqHAK28*	AUR62036631	476	3	54,185.71	6.8	Plasma membrane	Chr17: 53383724–53386568
*CqHAK29*	AUR62037170	741	8	82,669.67	6.92	Plasma membrane	Chr18: 11688609–11693135
*CqHAK30*	AUR62020155	745	9	83,356.3	8.66	Plasma membrane	Chr18: 31295458–31306880

## Data Availability

Data are available in the manuscript and in the Supplementary Materials.

## Supplementary Materials

The following supporting information can be downloaded at: https://www.mdpi.com/article/10.3390/plants12213747/s1, Figure S1: The sequence information of 10 conserved motifs of the *HAK* genes in quinoa, including the sequence logo and amino acids and the amino acid numbers of each motif; Figure S2: The Ka/Ks values of the *HAK* gene pairs for Cq-At, Cq-Cq, and Cq-Bv. Table S1: Details of the *HAK* genes from Arabidopsis, rice and *Beta vulgaris*; Table S2: Segmentally and tandemly duplicated *CqHAK* gene pairs; Table S3: The synteny gene pairs of *C. quinoa*, *A. thaliana*, and *B. vulgaris* as well as the Ka/Ks of comparative synteny gene pairs; Table S4: Detailed information on the expression levels (FPKM) of *CqHAK* genes retrieved from transcriptome data for *C. quinoa*; Table S5: Detailed information on the available expression levels (FPKM) of *CqHAK* genes response to various stress treatments retrieved from transcriptome data for *C. quinoa*; Table S6: Primers used in this study.
