# Supplementary material for: Genome-Wide Identification and Characterization of the HAK Gene Family in Quinoa (Chenopodium quinoa Willd.) and Their Expression Profiles under Saline and Alkaline Conditions
Source: Plants (Basel). 2023 Nov 1;12(21):3747. doi: 10.3390/plants12213747 (PMC10650088; doi:10.3390/plants12213747)
Supplement: Supplementary file 1 [file plants-12-03747-s001.zip › Figure S1.pdf]

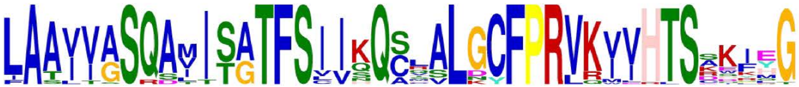
MEME-1 LAAVVASQAMISATFSIIKQSLALGCFPRVKVVHTSSKIEG 41

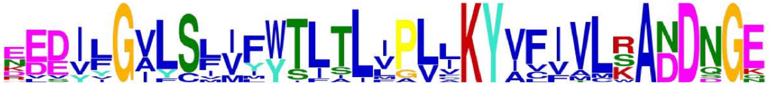
MEME-2 EEDILGVLSLIFWTLTLIPLJKYVFIVLRABDNGE 35

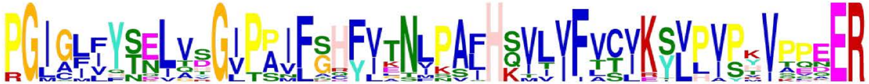
MEME-3 PGIGLFYSELVSGIPAIFSHFVTNLPAFHSVLVFVCVKSVPVPKVPPEER 50

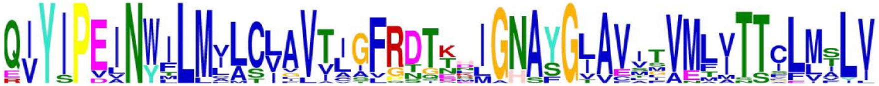
MEME-4 QIYIPEINWILMVLCLAVTJGFRDTKDIGNAYGJAVITVMLVTTCLMSLV 50

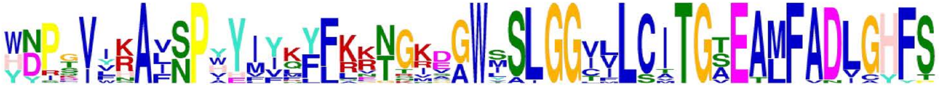
MEME-5 WBPGVIKAVSPYYIYKYFKKNGKDGWSSLGGVLLCITGTEAMFADLGHFS 50

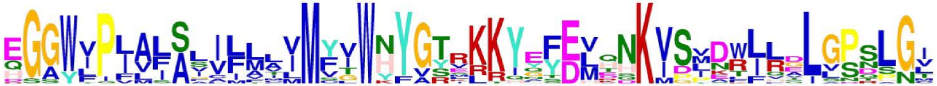
MEME-6 EGGWVPJALALILLLVMYVWNYGTRKKYEFELQNKVSVDWJLDLGPSLGI 50

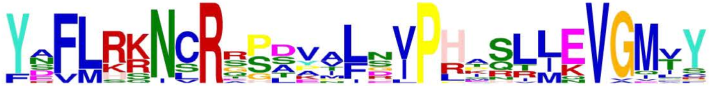
MEME-7 YAFLRKNCRRPDVALNVPHASLJEVGMVY 29

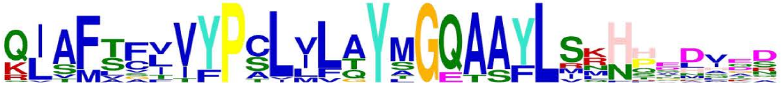
MEME-8 QJAFTFVVYPCLVLAYMGQAAYLSKHHEDVED 32

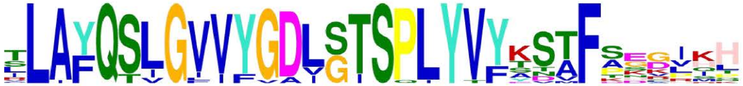
MEME-9 TLAYQSLGVVYGDLSTSPLYVYKSTFSEGIKH 32

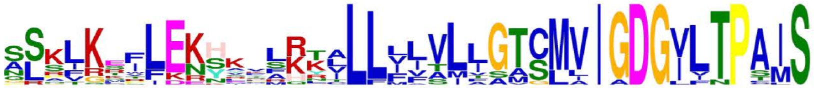
MEME-10 SSKLKEFLEKHKVLRKALLLJVLLGTCMVIGDGVLTPAIS 40

Figure S1. The sequence information of 10 conserved motifs of the *HAK* genes in quinoa, including the se-quence logo and amino acids and the amino acid numbers of each motif.
