# Supplementary figures and images for: Genome-Wide Identification and Characterization of the HAK Gene Family in Quinoa (Chenopodium quinoa Willd.) and Their Expression Profiles under Saline and Alkaline Conditions
Source: Plants (Basel). 2023 Nov 1;12(21):3747. doi: 10.3390/plants12213747 (PMC10650088; doi:10.3390/plants12213747)

# Ka/Ks value

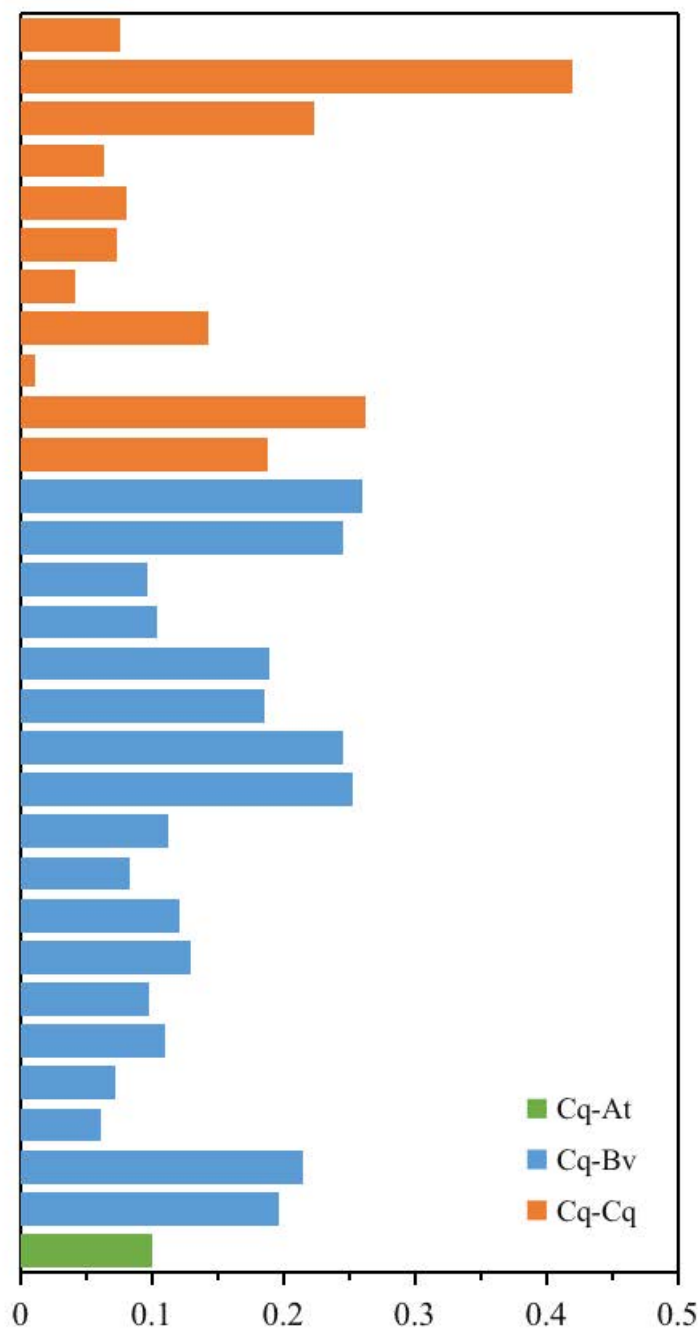

**Figure S2.** The Ka/Ks values of the *HAK* gene pairs for Cq-At, Cq-Cq, and Cq-Bv.

Supplement: Supplementary file 1 [file plants-12-03747-s001.zip › Figure S2.pdf]
